# A model-based analysis of phenytoin and carbamazepine toxicity treatment using binding-competition during hemodialysis

**DOI:** 10.1038/s41598-020-68333-3

**Published:** 2020-07-09

**Authors:** Vaibhav Maheshwari, Robert S. Hoffman, Stephan Thijssen, Xia Tao, Doris H. Fuertinger, Peter Kotanko

**Affiliations:** 1 Renal Research Institute, 315 E, 62nd St, New York City, NY 10065 USA; 2 Division of Medical Toxicology, Ronald O. Perelman Department of Emergency Medicine, NYU Grossman School of Medicine, New York, NY USA; 3 Global Research & Development, Fresenius Medical Care, Bad Homburg, Germany; 4 Department of Nephrology, Icahn School of Medicine at Mount Sinai, New York, USA

**Keywords:** Chemical biology, Computational biology and bioinformatics, Differential equations, Translational research, Applied mathematics, Toxicology

## Abstract

Hemodialysis (HD) has limited efficacy towards treatment of drug toxicity due to strong drug-protein binding. In this work, we propose to infuse a competitor drug into the extracorporeal circuit that increases the free fraction of a toxic drug and thereby increases its dialytic removal. We used a mechanistic model to assess the removal of phenytoin and carbamazepine during HD with or without binding-competition. We simulated dialytic removal of (1) phenytoin, initial concentration 70 mg/L, using 2000 mg aspirin, (2) carbamazepine, initial concentration 35 mg/L, using 800 mg ibuprofen, in a 70 kg patient. The competitor drug was infused at constant rate. For phenytoin (~ 13% free at t = 0), HD brings the patient to therapeutic concentration in 460 min while aspirin infusion reduces that time to 330 min. For carbamazepine (~ 27% free at t = 0), the ibuprofen infusion reduces the HD time to reach therapeutic concentration from 265 to 220 min. Competitor drugs with longer half-life further reduce the HD time. Binding-competition during HD is a potential treatment for drug toxicities for which current recommendations exclude HD due to strong drug-protein binding. We show clinically meaningful reductions in the treatment time necessary to achieve non-toxic concentrations in patients poisoned with these two prescription drugs.

## Introduction

Clinical recommendations often exclude the use of conventional hemodialysis (HD) for a majority of potentially toxic drugs, unless the toxicity is severe^1^. Even in cases of severe toxicity, HD is considered efficient only if the drug is not at all, or only weakly, bound to plasma proteins. The EXTRIP workgroup has provided guidelines for numerous toxins and recommended conventional HD as treatment option in only a handful of them, owing in part to the underlying fact that dialytic removal of strongly protein-bound drugs is poor^1–4^. Protein-binding results in a very small free fraction of the drug, and because only free solute can pass through the dialysis membrane pores, the diffusion gradient between blood and dialysate is too low. Increasing the free drug concentration in the extracorporeal circuit may render HD applicable for treatment, in which HD has been traditionally considered inefficient.

In this work, we propose a new method to treat protein-bound drug toxicity such that conventional HD is effective irrespective of degree of protein-binding. We infuse a competitor drug in the extracorporeal circuit. The competitor drug (*D*) will compete with toxic drug (*T*) for the same binding site on protein, and increase the free fraction of *T*, leading to its enhanced dialytic removal. Competitive binding provides significantly higher dialytic clearance of protein-bound uremic toxins in chronic HD patients^5^. The superiority of this proposed method over conventional HD, hemodiafiltration, and ideal membrane adsorption (ideal hemoperfusion) has been illustrated in computer simulations of intra-dialytic removal of protein-bound uremic toxins (PBUTs)^6^. Here we use a model-based approach to study the toxicity of two prescription drugs in conventional HD and competitive-binding augmented HD.

## Results

We simulated hemodialysis of a 70 kg man with the following compartment volumes: 3.5 L plasma, 14 L interstitial fluid, 28 L of intracellular fluid, and hematocrit of 35%. Initial albumin concentration was 4.3 g/dL (650 µM); the blood and dialysate flow rates were 250 and 500 mL/min, respectively; dialyzer specifications used were for an Optiflux F180NR dialyzer with a surface area of 1.8 m^2^. Conventional and free drug half-life for each drug is presented in Table 1.

**Table 1:** Conventional drug half-life and calculated free drug half-life.

Drug	Conventional half-life (t_1/2,total_)	Free drug half-life (t_1/2,free_)
Aspirin	20 min^7^	0.17 min
Carbamazepine	25 h^3^	1.25 h
Ibuprofen	2 h^8^	0.95 min
Phenytoin	35 h^2^	0.98 h

**Figure 1:**
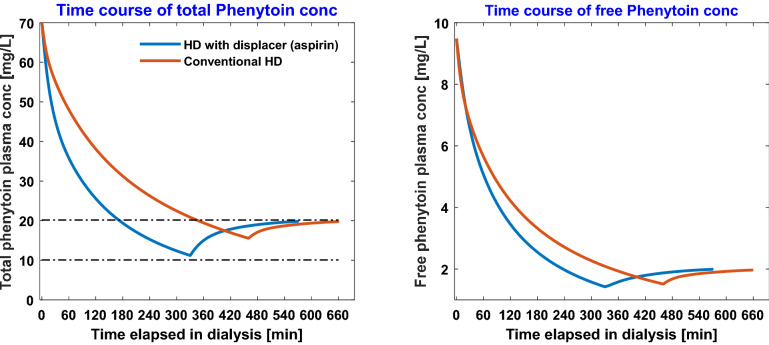
Time-course of total [left panel] and free [right panel] phenytoin during conventional hemodialysis vs. during competitive-binding augmented HD. In the latter, 2000 mg aspirin dissolved in 500 mL saline was infused at constant rate during the treatment session.

**Figure 2:**
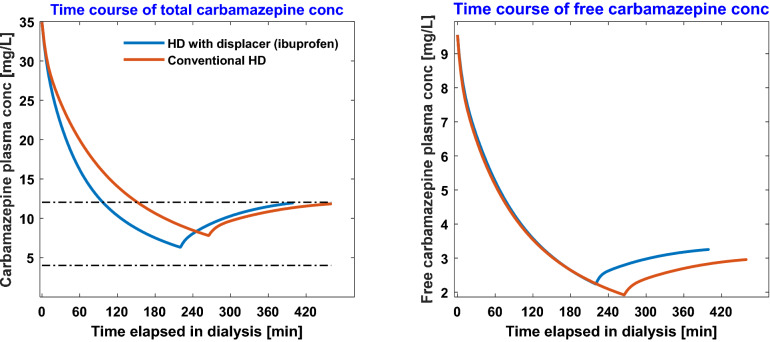
Time-course of total [left panel] and free [right panel] carbamazepine during conventional hemodialysis vs. during competitive-binding augmented HD. In the latter, 800 mg ibuprofen dissolved in 200 mL saline was infused at constant rate during the treatment session.

The time-course of the total and free serum phenytoin concentrations in conventional HD and HD with 2000 mg aspirin infusion is presented in Fig. 1. Starting from a serum phenytoin concentration of 70 mg/L, conventional HD requires 460 min to bring the patient within the therapeutic range, while HD with aspirin requires only 330 min. Similarly, the time-course of total and free serum carbamazepine concentration with 800 mg ibuprofen infused at a constant rate during conventional HD is shown in Fig. 2. Ibuprofen infusion reduces the time required to achieve a therapeutic range from 265 min of conventional HD to 220 min. Note that we continue to simulate the dialysis even when the serum drug concentration is well below maximum therapeutic concentration (MTC). This extra HD time accounts for post-dialytic rebound and keeps patient within therapeutic window (Figs. 1, 2).

Clearly, the degree of protein-binding defines the length of HD. In the case of carbamazepine, for which drug-protein binding is not very strong (~ 73% bound before dialysis), use of ibuprofen as binding competitor decreases the HD time by merely 45 min. On the other hand, when albumin-binding is strong, as in phenytoin (~ 86% bound before dialysis), constant aspirin infusion reduces the HD time by 130 min. In both scenarios, competitor drug infusion does not increase the free concentration of toxic drug beyond the initial free concentration of drug (Figs. 1, 2).

**Figure 3:**
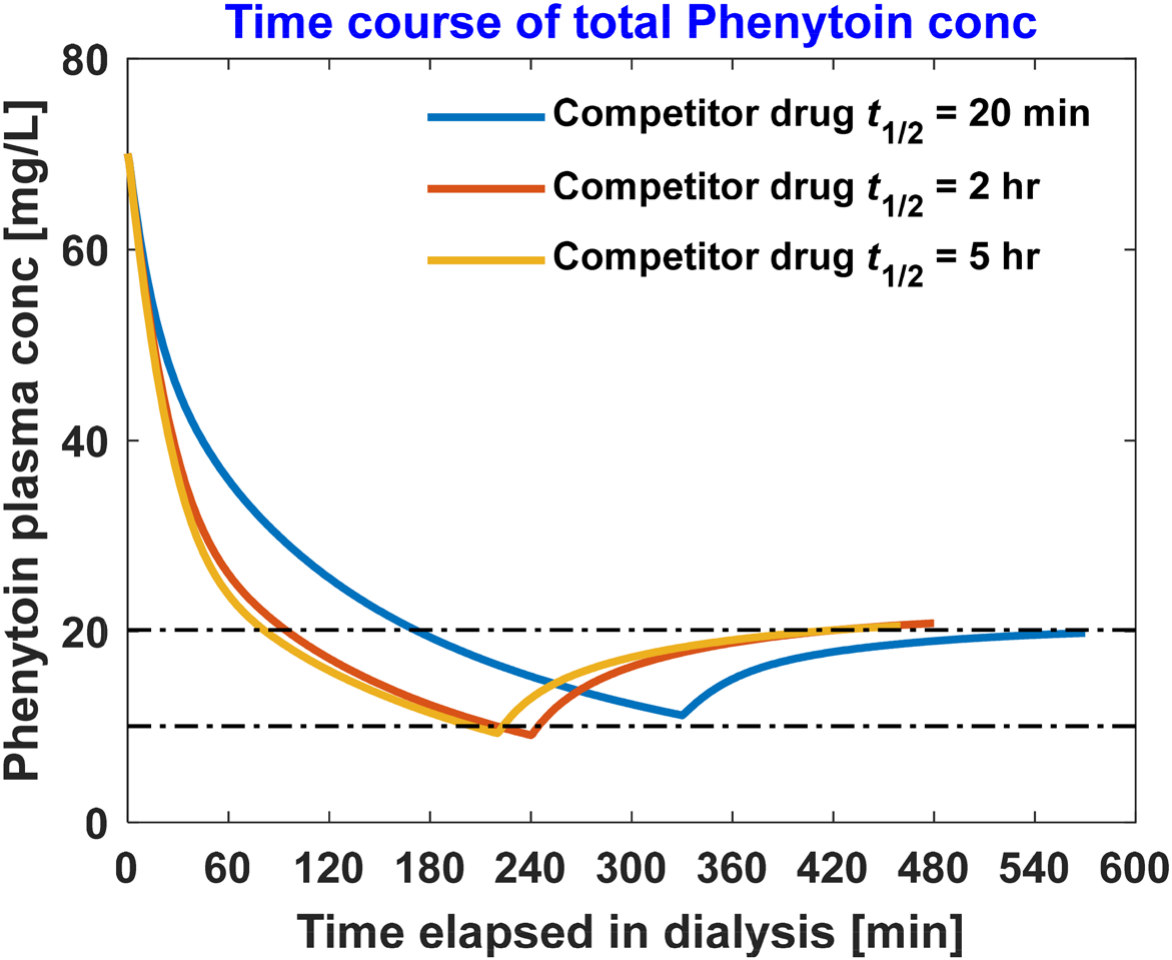
Effect of competitor drug half-life (t_1/2_) on the dialysis time to bring phenytoin within therapeutic window. Here, competitor drug binding affinity and dosage is kept same.

Competitor half-life plays an important role in reducing the HD time, e.g., in case of phenytoin, the competitor drug’s (aspirin) half-life is only 20 min; if another competitor drug with same binding affinity and same dosage, but with longer half-life is used, HD time reduces significantly even after accounting for post-dialytic rebound (Fig. 3). At the same time, having much longer half-life does not reduces the HD time further.

**Figure 4:**
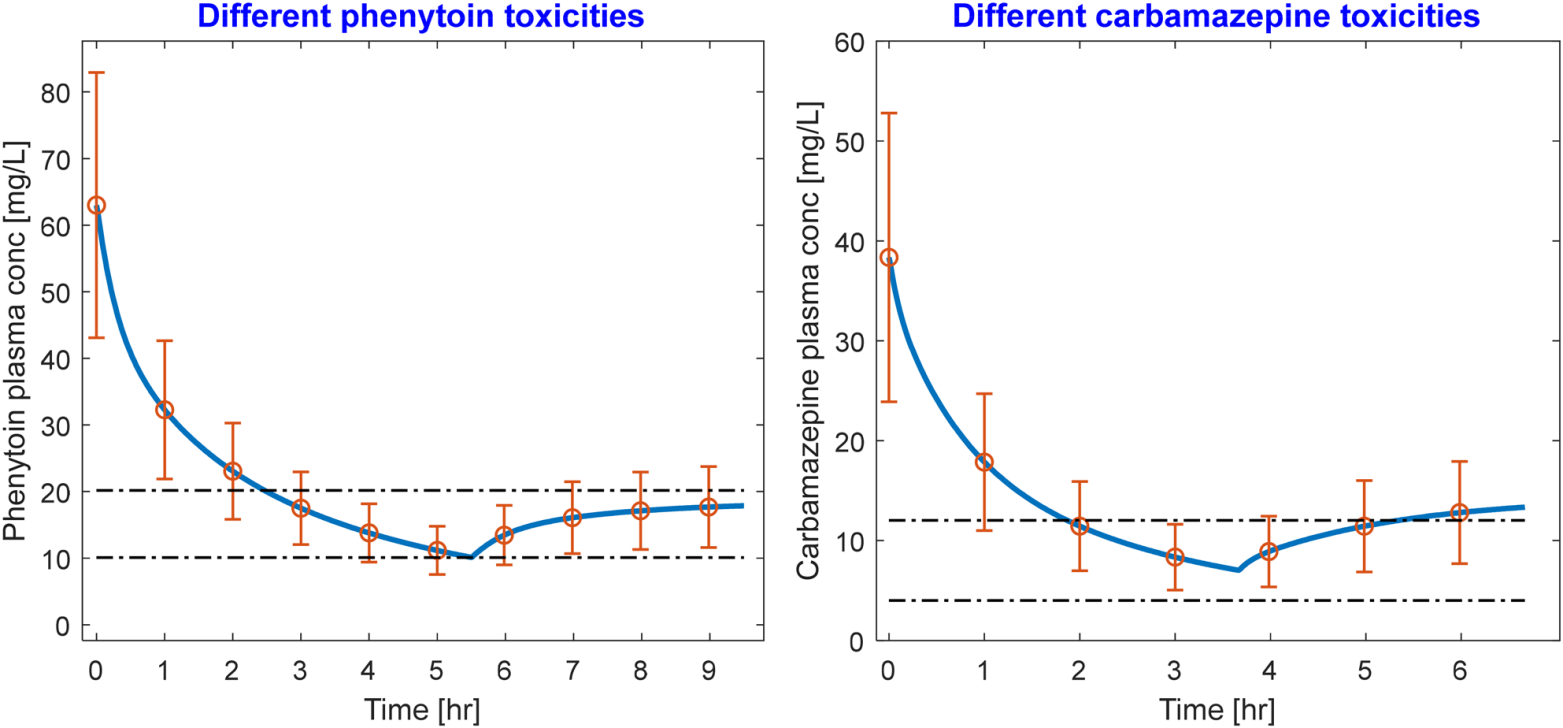
Time-course of phenytoin (left panel) and carbamazepine (right panel) toxicities, respectively, treated with 2000 mg of aspirin and 800 mg ibuprofen. In all toxicity scenarios, end-dialysis concentration is within therapeutic window. However, for higher pre-dialysis concentrations, post-dialytic rebound may be pronounced, suggesting need for higher competitor drug dose and/or longer treatment time.

To explore the pharmacokinetic variability in toxicity, we simulated an additional hypothetical patient of 70 kg weight with various toxicity levels, while keeping the competitor drug infusion dose same in all scenarios. We observed that irrespective of toxicity level, the phenytoin or carbamazepine concentration drops precipitously; however, for severe toxicities, post-dialytic rebound is pronounced to the extent that phenytoin and carbamazepine concentration is above MTC (Fig. 4). The rebound is more pronounced for carbamazepine (weakly bound), indicating significant sequestration of free carbamazepine in the inaccessible tissue compartments, which reflect in the form of post-dialytic rebound. To treat severe toxicities, increased dose of competitor drug or increasing the treatment duration may be viable options. Caution should be exercised while increasing the competitor drug dose beyond a certain limit, because it can also elicit deleterious effects in patients.

## Discussion

We present a new extracorporeal modality that may be potentially valuable to treat select drug toxicities. Phenytoin and carbamazepine are considered to illustrate the efficacy of the proposed method compared to conventional HD. A model-based approach was used to compare the two drug poisoning scenarios and potential treatment scenario using competitor drug. In both cases, the competitor drug reduced the required treatment time well below conventional HD time, with even a greater reduction in cases in which the drug protein-binding is stronger. The model was previously applied to protein-bound uremic toxins in which competitive binding outperformed both pre- and post-dilution hemodiafiltration and membrane adsorption^6,9^. It is important to note that HD results in a rapid decline in toxic drug concentration, which only refers to the plasma compartment. However, large amounts of the considered drug also exist in inaccessible pools, resulting in post-dialytic rebound. The reported HD time accounts for the rebound.

Model simulations suggest that competitor drug half-life plays an important role in the toxic drug removal. Along with free toxic drug, free competitor drug is also removed along the dialyzer; however, albumin-bound fraction of binding-competitor drug enters the systemic circulation with blood exiting from the dialyzer. This indicates that competition not only happens in dialyzer but also in patient’s drug compartments. As such, free competitor drug will diffuse into inaccessible fluid compartments and cause increase in free fraction of toxic drug which will diffuse into the blood compartment and then in the dialyzer, resulting in faster clearance of toxic drug. Longer half-life ensures that competitor drug has ample time to diffuse into inaccessible compartments and compete with toxic drug bound to protein. The effect of half-life is evident in the dialytic removal of phenytoin, where an increase in competitor drug half-life from 20 min to 2 h leads to further decrease in intra-dialytic time and still brings phenytoin within its therapeutic concentration (Fig. 3). Note that we modeled aspirin with a half-life of 20 min, however aspirin converts into salicylate which has half-life of 2 h and binds on Sudlow site I on albumin, and thus will compete with phenytoin. As such, the effective half-life of aspirin and metabolites that may compete with phenytoin is more than 20 min. Apparently, increasing competitor drug half-life beyond 2 h does not elicit further reduction in dialysis duration. Since the competitor drug competes with the drug in physiological compartments also, one may suggest that pre-dialysis intravenous infusion may even be better; however, this approach may elevate the free fraction beyond initial free fraction leading to larger apparent drug distribution volume and slower removal. More importantly, it is the free drug that has any clinical effect, thus we modeled that the competitor drug infusion should be extracorporeal and intra-dialytic.

In our analysis, we have assumed that drug half-lives, for both toxic and competitor drug, remain constant throughout the simulation period. In the beginning of HD when drug concentration is above the MTC – free concentration may be higher than the therapeutic free concentration; as such much of the free drug will diffuse into inaccessible compartments and thus remains unavailable for metabolism, resulting in longer half-life. Similarly, infusing competitor drug will result in higher levels of free drug in systemic circulation and it may affect the drug half-life, but we kept it constant in our simulations.

Competitive-binding technique can easily be extrapolated to treat toxicities in which the drug binds on two or more binding sites on protein, for e.g., to treat valproic acid (VPA) toxicity, in which VPA binds on both Sudlow Site I and II on albumin, one may need a competitor drug cocktail comprising, for e.g., aspirin and ibuprofen. We restricted our analysis to drugs that bind to one specific binding site and the competitor drug binds to the same binding site. The binding-competitor augmented HD can be superior to conventional HD or any other mode of extracorporeal therapy only if free fraction of the toxic drug at the initiation of HD is low. If toxicity is such that albumin binding sites are saturated and the initial free fraction is high, then infusing displacer(s) may only provide marginal gains. Competitive binding may still be beneficial in such a scenario only if competitor drug(s) is (are) infused optimally, for e.g., in the beginning of HD when free concentration is very high, no competitor drug should be infused; as HD progresses, free concentration of toxic drug drops precipitously (also observed in Figs. 1, 2), one may ramp up the competitor drug infusion. Optimal drug infusion can also be beneficial for treatment of severe toxicities (simulation results shown in Fig. 4), which result in high free drug concentration at the beginning of treatment and significant drug sequestration in inaccessible tissue compartment. The present model may be useful in obtaining an optimal infusion profile for the competitor drug such that toxic drug removal is maximized in a minimum HD time without increasing the competitor drug dosage. This aspect is beyond the scope of this work.

A careful risk assessment must be done before infusing a competitor drug because patient is already suffering from a drug overdose, and we intend to infuse additional exogenous substances. It should not be the case that synergistic effect of these two drugs is more harmful than the anticipated benefits, thus choice of competitor drug is important and drug-drug interaction should be considered a priori. The foremost requirement for the applicability of this method is that the infused drug should compete for the same binding site on albumin molecule where the toxic drug is bound. In the model simulations, we considered two intoxication scenarios where phenytoin binds on Sudlow site I and carbamazepine on Sudlow site II, i.e. ibuprofen binding on Sudlow site II will not be suitable for phenytoin removal. Another important aspect is the binding affinity. Ideally, the competitor drug binding affinity should be more than the intoxication drug being removed. However, if ideal displacer can only be infused in very small amount as per the clinical guidelines then competition will be ineffective. In such scenario, a binding competitor with weaker binding affinity in larger dose may be a better suited. Finally, the drug should be easily available for intravenous infusion.

Competitive-binding augmented HD is not only an effective technique to treat drug toxicity, but it is also easy to implement and inexpensive. Ease of implementation is a very important aspect because state-of-the-art extracorporeal modality for strongly bound drug toxicity, such as hemoperfusion, is sometimes inaccessible due to unavailability of adsorbent cartridge and their limited shelf life^10^. In addition, the adsorption capacity of hemoperfusion cartridges decline rapidly due to saturation^11^.

In conclusion, we propose a new method to treat protein-bound drug toxicity that renders conventional HD itself as preferred extracorporeal method irrespective of degree of protein-binding. Unlike conventional HD, in which separation is primarily driven by passive diffusion, the proposed method increases the magnitude of passive diffusion by way of reactive separation. Irrespective of drug protein-binding characteristics, the competitive binding during HD can essentially be applied to all drug toxicities in which traditionally hemoperfusion or plasmapheresis are generally preferred. The present model can be employed to test any binding-competitor drug and rank them according to their efficacy for specific toxicity.

## Material and methods

**Figure 5:**
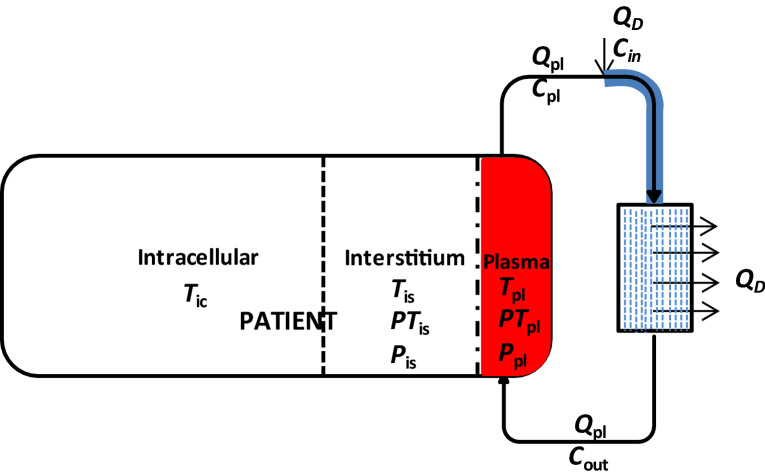
Schematic of patient-dialyzer system. A competitor drug is infused in the extracorporeal circuit to elicit competitive binding—free toxic drug as well as free competitor drug is removed along the fiber. All the infused volume is also removed along the fiber length, i.e. patient’s compartmental fluid volume does not change during dialysis.

We chose phenytoin and carbamazepine to illustrate the binding-competition during dialysis treat prescription drug toxicity. Below we describe our mathematical model for a toxic and a competitor drug, whereby both bind on the same binding site on albumin (carrier protein). The presented patient-dialyzer model system comprises three sub-models: (1) a three-compartment patient model, (2) an arterial tube segment model in which competitor drug is infused, and (3) a spatiotemporal model of the dialyzer in which a fraction of both toxic and competitor drug is removed. This model system was implemented in MATLAB 2019b. The model has previously been described in detail for PBUTs^6^. A fundamental difference between PBUTs and protein-bound drugs is that former are endogenous metabolic products continuously generated while latter are exogenous substances continuously metabolized. A block diagram of the complete model is shown in Fig. 5. In the following, we briefly describe each sub-model and the corresponding model assumptions.

### Patient model

It is assumed that both the toxic drug and the competitor drug distribute in body fluid—represented by three-compartment model of the patient: plasma pool, interstitial pool, and intracellular pool. Free drug being a small-sized molecule is equilibrated among all three compartments, while the protein-bound drug is restricted to extracellular compartment only, in which both drugs bind to albumin (*P*). Extracellular space is sub-divided into plasma and interstitial pool because of unequal protein concentration between them. The toxic drug (*T*) and the competitor drug (*D*), both share the same binding site on albumin. The dynamic equilibrium among *T*, *D*, and *P* is depicted by the reaction scheme below.

$$\begin{array}{*{20}c} {P + T\mathop \Leftrightarrow \limits^{{ K_{A,T } }} PT ; \;K_{A,T} = \frac{{a_{1} }}{{a_{2} }}} \\ {P + D\mathop \Leftrightarrow \limits^{{ K_{A,D } }} PD ;\; K_{A,D} = \frac{{d_{1} }}{{d_{2} }}} \\ \end{array}$$

Generic mass balance applicable to free drugs (*T*, *D*), protein-drug complexes (*PT*, *PD*), and free protein (*P*) in the plasma compartment is shown in Eq. (1).

1 $$\begin{aligned} & \frac{{d\left( {V_{pl} C_{pl} } \right)}}{dt} = - Q_{pl} C_{pl} + Q_{pl} C_{out} + \left( {1 - \emptyset_{C} } \right)K_{ip} \left( {C_{is} - C_{pl} } \right) + \left( { - r_{C,pl} } \right)V_{pl} - \left( {1 - \emptyset_{C} } \right)\lambda_{C} C_{pl} V_{pl} , \\ & C \in \left\{ {T,PT,D,PD,P} \right\}; \emptyset_{C} = \left\{ {\begin{array}{*{20}c} {0, if C \in \left\{ {T,D} \right\} } \\ {1, ifC \in \left\{ {PT,PD,P} \right\}} \\ \end{array} } \right. \\ & e.g., \frac{{d\left( {V_{pl} T_{pl} } \right)}}{dt} = - Q_{pl} T_{pl} + Q_{pl} T_{out} + K_{ip} \left( {T_{is} - T_{pl} } \right) + \left( { - a_{1} P_{pl} T_{pl} + a_{2} PT_{pl} } \right)V_{pl} - \lambda_{T} T_{pl} V_{pl} \\ \end{aligned}$$

Here $C_{pl}$ is the solute concentration in plasma entering the extracorporeal circuit, $C_{out}$ is the solute concentration in the post-dialyzer stream going back in the patient, $C_{is}$ is solute concentration in interstitial pool; $V_{pl}$ is plasma volume; $Q_{pl}$ is plasma flow rate; $K_{ip}$ is free solute mass-transfer coefficient between interstitial and plasma pool; $- r_{C,pl}$ is the reaction rate accounting for rate of solute appearance and disappearance in plasma; $a_{1}$, $d_{1}$ are association constants for toxic and competitor drug, respectively, while $a_{2}$, $d_{2}$ are respective dissociation constants in protein-drug binding; and *λ*_*C*_ accounts for first order elimination of both drugs. Additional details regarding endogenous drug elimination are provided in the section “Drug half-life”. Unlike chronic HD patients, we assume that the poisoned patient will have native kidney function, unless there is acute kidney injury induced by toxicity. The intact kidneys will eliminate both toxic and competitor drugs from plasma in tandem with HD. However, we do not model drug elimination by the kidneys or liver, rather we lump all the endogenous losses in the drug half-life. This modeling approach adheres to half-life definition without itemizing different modes of drug elimination from systemic circulation. One underlying modeling assumption is that we used drug half-life data gathered in healthy subjects i.e. toxicity did not affect the drug metabolism or elimination in the patient. Additionally, the half-life of both drugs is assumed to be constant during the simulation period.

Generic mass balance of all the species in the interstitial pool is expressed in Eq. (2). We neglected the effect of lymphatic transport on protein or protein-solute exchange between interstitial and plasma pool.

2 $$\begin{aligned} \frac{{d\left( {V_{is} C_{is} } \right)}}{dt} & = - \left( {1 - \emptyset_{C} } \right)K_{ip} \left( {C_{is} - C_{pl} } \right) + \left( {1 - \emptyset_{C} } \right)K_{ic} \left( {C_{ic} - C_{is} } \right) + \left( { - r_{C,is} } \right)V_{is};C \in \left\{ {T,PT,D,PD,P} \right\} \\ e.g., \;\frac{{d\left( {V_{is} T_{is} } \right)}}{dt} & = - K_{ip} \left( {T_{is} - T_{pl} } \right) + K_{ic} \left( {T_{ic} - T_{is} } \right) + \left( { - a_{1} P_{is} T_{is} + a_{2} PT_{is} } \right)V_{is} . \\ \end{aligned}$$

Solute mass balance in intracellular pool is given by Eq. (3). No albumin exists in intracellular space thus Eq. (3) is applicable for free solutes only. Note, the absence of intracellular binding protein allows us to omit the reaction rate terms in the intracellular solute mass balance.

3 $$\frac{{d\left( {V_{ic} C_{ic} } \right)}}{dt} = - K_{ic} \left( {C_{ic} - C_{is} } \right);C \in \left\{ {T,D} \right\}$$

Here, $K_{ic}$ is the free drug diffusive mass-transfer coefficient between intracellular and interstitial pool; $V_{is}$ and $V_{ic}$, respectively, are interstitial and intracellular fluid volume; $- r_{C,is}$ is solute reaction rate term in interstitial pool. We assumed a $K_{ip}$ of 1,200 mL/min and $K_{ic}$ of 100 mL/min for the simulations for both *T* and *D*—values were adapted from^9^. A higher value of $K_{ip}$ than $K_{ic}$ indicate a more permeable structure of the capillary endothelium compared to the cellular walls. Note that we assumed same $K_{ip}$ and same $K_{ic}$ for both drugs since both are of comparable size and inter-compartmental diffusive mass transfer is primarily dependent on molecular size. Unlike conventional HD patients with end-stage kidney disease, there is no fluid removal from the poisoned subject. Intra-dialytic fluid gain is also assumed to be zero. Hence, we do not consider the volume balance for different compartments; further, we assume that infused competitor drug fluid volume is removed along the fiber length.

### Arterial tube-segment model

Once blood leaves the patient and enters the extracorporeal circuit, the competitor drug (*D*) is infused at constant rate $Q_{D}$ into the arterial tube segment. The drug ‘*D*’ competes with the toxin ‘*T*’ bound on albumin ‘*P*’. Since drug infusion is accompanied with volume, the model also accounts for dilution and its effect on binding equilibrium between *D*, *T*, and *P*. Axial diffusion in the tube-segment is neglected due to negligible diffusion coefficient of drugs in the direction of flow ^12^. In Eq. (4), we present the generic sub-model explaining the shift in the protein-drug dynamic equilibrium between infusion site and dialyzer blood inlet, i.e. along the highlighted tube segment (Fig. 5).

4 $$\begin{aligned} Q_{tube} & = Q_{pl} + Q_{D}; \frac{{\partial C_{tube} }}{\partial t} = - \frac{{Q_{tube} }}{{A_{tube} }}\frac{{\partial C_{tube} }}{\partial x} + \left( { - r_{C,tube} } \right);C \in \left\{ {T,PT,D,PD,P} \right\}; \\ e.g. \frac{{\partial P_{tube} }}{\partial t} & = - \frac{{Q_{tube} }}{{A_{tube} }}\frac{{\partial P_{tube} }}{\partial x} + \left( {\left( { - a_{1} P_{tube} T_{tube} + a_{2} PT_{tube} } \right) + \left( { - d_{1} P_{tube} D_{tube} + d_{2} PD_{tube} } \right)} \right) \\ \end{aligned}$$

Boundary conditions for the tube model are given below.

5 $$\left. {C_{x} } \right|_{t = 0} = 0, \left. {C_{t} } \right|_{x = 0} = \frac{{Q_{pl} C_{pl} + Q_{D} C_{inf} }}{{Q_{tube} }}$$

$C_{pl}$ and $C_{inf}$ in Eq. (5) denote the concentration of the solute, respectively, in plasma and in infusion stream. In infusion stream, only the free drug exists, thus $C_{inf}$ is zero for all other species, except for *D*. At the end of tube-segment i.e. at dialyzer inlet, *T*, *D*, and *P* achieve new equilibrium in which toxic drug free concentration is increased.

### Dialyzer model

For the dialyzer model, we assumed that blood entering the dialyzer is equally distributed among all fibers (*N*). Hence, it suffices to consider the mass balance along one fiber only. Similarly, the dialysate flowing in counter-current to blood is also assumed to distribute equally in the interstitial space around the fibers. Along the fiber, both *T* and *D* are removed by diffusion and convection; the latter is present due to the removal of fluid volume which was infused in the arterial tube-segment. It is assumed that the fluid removal rate along the dialyzer is uniform, i.e. plasma flow rate linearly decreases, while dialysate flow rate linearly increases in counter-current direction (Eq. (6)).

6 $$Q_{p} \left( x \right) = Q_{tube} - \frac{x}{L}Q_{D} , \,Q_{d} \left( x \right) = Q_{di} + \frac{L - x}{L}Q_{D} .$$

Solute mass balance in dialyzer results in the following generic model for the plasma and dialysate side (Eq. (7)).

7 $$\begin{aligned} \frac{{\partial C_{p} }}{\partial t} & = - \frac{1}{N \cdot A}\frac{{\partial \left( {Q_{p} C_{p} } \right)}}{\partial x} + \frac{1}{N \cdot A}\frac{{\partial Q_{p} }}{\partial x}C_{p} \left( {1 - \sigma_{C} } \right) - \frac{Pe}{{e^{Pe} - 1 }}\frac{1}{N \cdot A \cdot L}K_{o} A \left( {C_{p} - C_{d} } \right) + \left( { - r_{C,p} } \right), \\ \frac{{\partial C_{d} }}{\partial t} & = \frac{1}{{N \cdot A_{d} }}\frac{{\partial \left( {Q_{d} C_{d} } \right)}}{\partial x} - \frac{1}{{N \cdot A_{d} }}\frac{{\partial Q_{d} }}{\partial x}C_{p} \left( {1 - \sigma_{C} } \right) + \frac{Pe}{{e^{Pe} - 1 }}\frac{1}{{N \cdot A_{d} \cdot L}}K_{o} A \left( {C_{p} - C_{d} } \right) + \left( { - r_{C,d} } \right), \\ C & \in \left\{ {T,PT,P, D, PD} \right\} \\ \sigma_{C} & = \left\{ {\begin{array}{*{20}c} { 0 , if\; C \in \left\{ {T,D} \right\} } \\ {0.999, if \; C \in \left\{ {PT,PD,P} \right\}} \\ \end{array} } \right.;\;K_{o} A = \left\{ {\begin{array}{*{20}c} {800 , \,\,\;if\; C \in \left\{ {T,D} \right\} } \\ { 0 , \,\;\;if\;C \in \left\{ {PT,PD,P} \right\}} \\ \end{array} \,{\text{mL}}/\min .} \right. \\ \end{aligned}$$

Here $C_{p}$ and $C_{d}$ are the solute concentration in blood and dialysate side stream varying along axial position and time; $Q_{p}$ and $Q_{d}$ denote plasma and dialysate flow rate in the dialyzer; $N$ is the number of fibers in dialyzer casing; $A$ and $A_{d}$, respectively, are inner cross-section area of a fiber and cross-section area of the interstitial space around a fiber; ${\text{K}}_{{\text{o}}} {\text{A}}$ is the diffusive mass transfer coefficient for free solutes; $\sigma_{C}$ is the membrane reflection coefficient. In the presence of convection, the diffusive exchange of free solute is adjusted by a function of Péclet number (*Pe*)—defined as ratio of the convective mass transfer rate to the diffusive mass transfer rate. Many drugs depict affinity towards two bindings sites: primary and secondary binding site, as in case of phenytoin^13^. In the model presented here, we have neglected the secondary binding site contribution because majority of drug is bound to primary binding site only.

#### Drug half-life

The drug half-life reported in the literature corresponds to total serum concentration i.e. after one half-life, the total serum concentration is half of the peak serum concentration. This definition does not differentiate between free and bound fraction of the drug. Physiologically it is always the free drug that metabolizes; subsequently bound drug frees from protein and appears in the systemic circulation owing to shift in protein-drug binding equilibrium and diffusion from inaccessible pools. To account for this physiological aspect in our model, we adjust the free drug half-life ($\lambda_{C}$) such that it conforms to the conventional drug half-life definition. We used the same three-compartment patient model and assumed that at peak drug concentration, drug is equilibrated in plasma, interstitial, and intracellular pool.

We simulated two scenarios: (A) Phenytoin toxicity with an initial serum concentration 70 mg/L (therapeutic range 10–20 mg/L^2^), treated with 2000 mg acetylsalicylic acid (aspirin) dissolved in 500 mL saline, (B) Carbamazepine toxicity with an initial serum concentration of 35 mg/L (therapeutic range 4–12 mg/L^3^), treated with 800 mg ibuprofen dissolved in 200 mL saline. Phenytoin and aspirin bind on Sudlow Site I with respective binding affinities of 1.56 × 10^4^ M^−1^ and 1.90 × 10^5^ M^−1^^14,15^. On the other hand, carbamazepine and ibuprofen bind on Sudlow Site II with respective binding affinities of 4.9 × 10^3^ M^-1^ and 1.76 × 10^5^ M^−1^ ^16,17^. Here, phenytoin and carbamazepine binding affinities were calculated using their therapeutic concentrations: 20 mg/L for phenytoin with 90% bound and 12 mg/L for carbamazepine with 75% bound, and 4.3 g/dL serum albumin. Drug association constant ($$a_{1}$$ or $$d_{1}$$) was assumed to be 10^8^ M^−1^ min^−1^ ^18^, while dissociation constant was calculated using binding affinity definition $$K_{A,toxin} = {\raise0.7ex\hbox{${a_{1} }$} \!\mathord{\left/ {\vphantom {{a_{1} } {a_{2} }}}\right.\kern-\nulldelimiterspace} \!\lower0.7ex\hbox{${a_{2} }$}}$$ or $$K_{A,displacer} = {\raise0.7ex\hbox{${d_{1} }$} \!\mathord{\left/ {\vphantom {{d_{1} } {d_{2} }}}\right.\kern-\nulldelimiterspace} \!\lower0.7ex\hbox{${d_{2} }$}}$$.
